# Rheumatoid Arthritis and miRNAs: A Critical Review through a Functional View

**DOI:** 10.1155/2018/2474529

**Published:** 2018-03-29

**Authors:** Maria Cristina Moran-Moguel, Stefania Petarra-del Rio, Evangelina E. Mayorquin-Galvan, Maria G. Zavala-Cerna

**Affiliations:** ^1^Molecular Biology and Genomics Department, Centro Universitario de Ciencias de la Salud, Universidad de Guadalajara, Guadalajara, JAL, Mexico; ^2^Immunology Research Laboratory, Immunology Division, Programa Internacional de Medicina, Universidad Autónoma de Guadalajara, Guadalajara, JAL, Mexico

## Abstract

Rheumatoid arthritis (RA) is a systemic autoimmune disease with severe joint inflammation and destruction associated with an inflammatory environment. The etiology behind RA remains to be elucidated; most updated concepts include the participation of environmental, proteomic, epigenetic, and genetic factors. Epigenetic is considered the missing link to explain genetic diversification among RA patients. Within epigenetic factors participating in RA, miRNAs are defined as small noncoding molecules with a length of approximately 22 nucleotides, capable of gene expression modulation, either negatively through inhibition of translation and degradation of the mRNA or positively through increasing the translation rate. Over the last decade and due to the feasibility of the identification of miRNAs among different tissues and compartments, they have been proposed as biomarkers for diagnosis, prognosis, and response to treatment in different pathologies. Nevertheless, miRNAs seem to be important regulators of networks instead of single genes; their hypothetical use as biomarkers needs to rely on a functional integrative description of their effects in the biological process of autoimmune conditions which until now is missing. Therefore, we underwent a bibliographic search for review and original articles related to miRNAs and their possible implications in rheumatoid arthritis. We found 48 different studies using the key words “miRNAs” or “micro-RNAs” and “rheumatoid arthritis” with restriction of publication dates from 2011 to 2016, in humans, using the English language. After a critical reading, we provide in this paper a functional view with respect to miRNA biogenesis, interaction with targets that are expressed in specific cells and tissues, during different stages of inflammatory responses associated with RA, and recognized specific areas where miRNAs might also have a pathogenic role but remain undescribed. Our results will be useful in designing future research projects that can support miRNAs as biomarkers or therapeutic targets in RA.

## 1. Introduction

Rheumatoid arthritis (RA) is a systemic autoimmune disease with risk of function disability due to articular damage as a consequence of ongoing important inflammation, which is irretrievable. Rheumatoid joints exhibit an inflammatory environment that favors the activation of neutrophils, T cells, B cells, macrophages, osteoclasts, and synovial fibroblasts [1]. These cells maintain a crosstalk through the production of cytokines, and their activation induces the secretion of enzymes and other products that contribute to the destruction of cartilage and bone tissues [2]. AR etiology remains to be completely elucidated, but some authors suggest that it is the result of a combination of genetic and environmental factors that eventually converge in an overreactive immune system [3]. Even though several genes have been identified as genetic factors that contribute to RA susceptibility, such as *HLA-DR*, *PTPTN22*, *CTLA-4*, and *PADI4*, we still do not have a clear picture of how these genetic factors induce the appearance of RA [4]; it rather seems to be a polygenic disease, where a combination of gene mutations might be needed for disease presentation. Other factors such as epigenetics are considered missing links to explain inconsistencies in associations between genetic polymorphisms in candidate genes and disease presentation [5].

### 1.1. Discovery and Biogenesis of miRNAs

**Figure 1:**
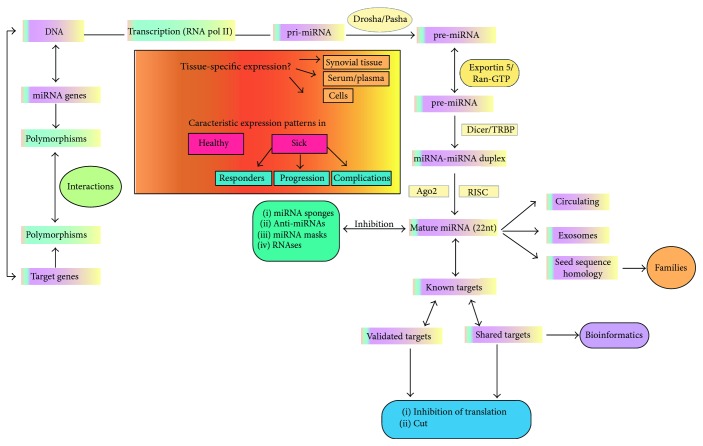
miRNA biogenesis, functions, and special considerations for their analysis.

In humans, protein-coding sequences occupy approximately up to 1.5% of the genome, and when considering intervening sequences (introns and 3′ or 5′ UTRs), the number increases approximately to 28%; the rest of the genetic material was considered “junk” DNA, until 2012, when it was calculated that almost 80% of the genome participates in biochemical activities functionally important for DNA expression [6] and 70% of the DNA has the capacity to produce noncoding RNA (ncRNAs) [7]. Within these, we identify micro-RNAs (miRNAs), which are defined as small molecules, approximately 22 nucleotides (nt) in length; their function is to modulate gene expression either negatively through inhibition of translation and direct degradation of mRNA or positively by increasing the translation rate [8]. Biogenesis of miRNA genes initiates with their transcription by RNA polymerases II, which gives rise to monocistronic or polycistronic long primary transcripts named primary miRNA or pri-miRNA, with a length of 200 nt to several kilobases, folded into hairpin structures containing imperfectly base-paired stems. In the cell nucleus, pri-miRNAs are processed by the multimeric protein complex Drosha (type III RNase) and the DiGeorge syndrome critical region gene 8 (*DGCR8*) into a shorter pre-miRNA, which is 70–100 nt [9]. Pre-miRNA then is transported into the cytoplasm of the cell by exportin 5, where another RNase III known as Dicer, along with human immunodeficiency virus transactivating response RNA-binding protein (TRBP), cleaves it into a smaller molecule (21 nt long), giving rise to a miRNA: miRNA duplex. The duplex is unwound by a helicase, and the miRNA “guide” strand is incorporated into the multiprotein RNA-induced silencing complex (RISC) containing Argonaut proteins, then they can migrate to find their targets and regulate their transcription [10, 11]. There are up to four different types of Argonauts (Ago); nevertheless, Ago2 has been consistently implicated with miRNA function and mRNA degradation by its endonuclease activity [12]. Therefore, Ago2 is important for two actions: provides a functional miRNA and effectively participates in target mRNA degradation [8]. It is estimated that miRNA genes constitute approximately 1–2% of the entire genome and that they could potentially regulate up to 30% of codifying genes. The genes that codify for miRNAs are located throughout the chromosomes, except for the Y chromosome; miRNA genes are predominantly identified in introns (70%) and in cancer-associated genomic regions (52.5%) [13]. In Figure 1, we describe miRNA biogenesis functions and special considerations for their analysis.

Transcription of miRNA genes by RNA polymerases II arises as primary miRNA (200 nt length), after being processed by Drosha (type III RNase) and DGCR8 (DiGeorge syndrome critical region gene 8); they are transformed into pre-miRNA (70–100 nt long). Pre-miRNA then is transported into the cytoplasm by exportin 5, where another RNase III known as Dicer, along with TRBP, cleaves it into a smaller molecule (21 nt long), giving rise to a miRNA: miRNA duplex. The duplex is unwound by a helicase, and the miRNA guide strand is incorporated into the multiprotein RNA-induced silencing complex (RISC) containing Argonaut proteins (Ago2), which is capable of migrating, and effectively participates in target mRNA regulation or degradation. miRNAs are easily found in body fluids due to their presence in the complex with proteins or in macrovesicles that prevent their degradation by RNases, which confers them the possibility to migrate into different tissues. For a miRNA to work as an inhibitor of mRNA translation, it requires the presence of a seed sequence (6 nt long), which holds the complementarity region with its target mRNA; only after binding of the seed sequence can miRNAs effectively inhibit mRNA translation. The presence of conserved seed sequences among different miRNAs has made possible the identification of families. More than 1000 different miRNAs have been described so far, which made evident the necessity of bioinformatics tools to manage the extremely high flow of new miRNA-related data; however, validating a possible miRNA target is a challenging task and should be performed before putative roles for miRNAs in human diseases.

### 1.2. Attributable Functions of miRNAs

For each miRNA, single or multiple mRNA targets have been described, which can participate in distinct processes, such as immune responses, cellular differentiation, cellular proliferation, metabolism, homeostasis, apoptosis, malignant transformation of cells, and senescence [14]. For miRNAs to induce an effective inhibition, the presence of a “seed” sequence (six nucleotides long) is required, due to complementarity-dependent binding. Seed sequences have also been used to identify conserved sequences among miRNAs and therefore to constitute families [15]; a small number of miRNAs can directly bind to proteins [10], but the participation of the seed sequence in this process is unknown. More than 1000 different miRNAs have been described so far, which are the focus of extensive research in several conditions, such as RA [12, 16]. More than often, miRNAs are postulated as biomarkers due to their presence in body fluids and cells, relatively easy measurement techniques, and the fact that they are contained in macrovesicles that prevent their degradation by RNases [17]. Their use as biomarkers could aid in early diagnosis, to monitor disease course, prognosis, and response to treatment.

In the last few years, bioinformatics tools have been developed to manage the extremely high flow of miRNA-related new data; miRBase, for example, contains 1872 human miRNA precursors that produce 2578 different mature miRNAs [10]. Target prediction algorithms are evolving in parallel which allow computer programs to use information collected from previously described and verified miRNAs to identify new ones; however, validating a possible miRNA target is a challenging task, since it is expensive and time consuming, considering that each miRNA has several potential targets.

Bioinformatics tools can serve distinct purposes, including discovery of new miRNAs, target prediction, and analysis of complementarity. An important consideration with respect to miRNA research has been the use of different nomenclatures by several authors [10]. Additionally, the term *3p* or *5p* at the end refers to mature forms of miRNAs; several studies fail to specify whether their results refer to mature miRNAs. We therefore used the information as it was presented by the authors and tried to unify nomenclatures whenever possible; nevertheless, this is an important consideration that should be included in future research studies of their participation in human diseases.

### 1.3. Implications of miRNAs in Rheumatoid Arthritis

In 2007, antibodies against GW/P bodies were identified in RA and Sjögren syndrome patients. GW/P bodies are cellular compartments that contain a glycine/tryptophan- (G/W-) rich mRNA-binding protein GW182. They represent sites for mRNA processing and degradation and are important in the RNA interference pathway [18]. Later, three different studies reported dysregulation in miRNA expression when peripheral blood [19] and synovium [20, 21] from RA patients were evaluated. The abnormal expression of miRNAs in RA patients has been documented in more than a dozen studies, most of them centered around T cell differentiation (Th17), the production of inflammatory cytokines, and B cell activation. Furthermore, some miRNAs have different patterns of expression during the disease course, allowing identification of disease activity. In the last decade, the use of new biologic drugs to treat RA has led to unraveling of mechanisms behind the generation of inflammatory responses, and these, along with miRNAs that regulate their expression, might be used to become candidate biomarkers in RA [12]. Autoantibodies against citrullinated antigens (ACPA) are considered important in the development of the disease, even identified before the onset of RA; a recent study suggested a correlation between miR-146a and the presence of ACPA in patients with periodontal disease, an inflammatory condition within oral cavities, with evidence of local citrullination and therefore a possible inductor of autoantibody production [12]. However, the possibility that this miRNA is associated with regulation of targets that will codify for proteins associated with this process of citrullination remains undescribed and should lead to further investigations. Despite this information, there are several limitations that need to be fully clarified before miRNAs are used as biomarkers in RA; first, we have conflicting information in miRNA patterns of expressions in different tissues; for example, miR-132 and miR-155 can be found upregulated in PBMC but downregulated in plasma, making evident the dynamics of miRNAs through different compartments [3]. Second, there is a limited number of miRNAs identified as dysregulated in the periphery that are capable of differentiating RA from osteoarthritis (OA), a degenerative condition that affects the joints. Both pathologies share the feature of articular inflammation, and the possibility of these miRNAs being related to inflammatory conditions instead of the pathology itself exists. Finally, because miRNAs seem to be important regulators of networks instead of single genes, their hypothetical use as biomarkers needs to rely on a functional integrative description of their effects in the biological process of autoimmune conditions, which until now is missing. In order to obtain more information that can be used to guide future research studies for the clarification of miRNA participation as biomarkers in RA, we underwent a bibliographic search for review and original articles related to miRNAs and their possible implication in rheumatoid arthritis, and instead of enumerating miRNAs which has been done extensively in the past, we highlighted their functional properties with respect to their targets and identified areas where research is needed, to support miRNAs as biomarkers or therapeutic targets.

## 2. Methods

We performed a search on MEDLINE, which is the primary component of PubMed®, part of the Entrez series of databases provided by the US National Library of Medicine National Center for Biotechnology Information (NCBI). We used the key words “miRNAs” AND “rheumatoid arthritis”, and a second search was performed using the key words “micro-RNAs” AND “rheumatoid arthritis.” We restricted results to papers published from 2011 to 2016, studies with results in humans and published in English. Articles were screened for their relevance concerning miRNA participation in RA and excluded studies about other autoimmune diseases. Supplementary material concerning RA pathophysiology was included when necessary.

## 3. Results and Discussion

**Figure 2:**
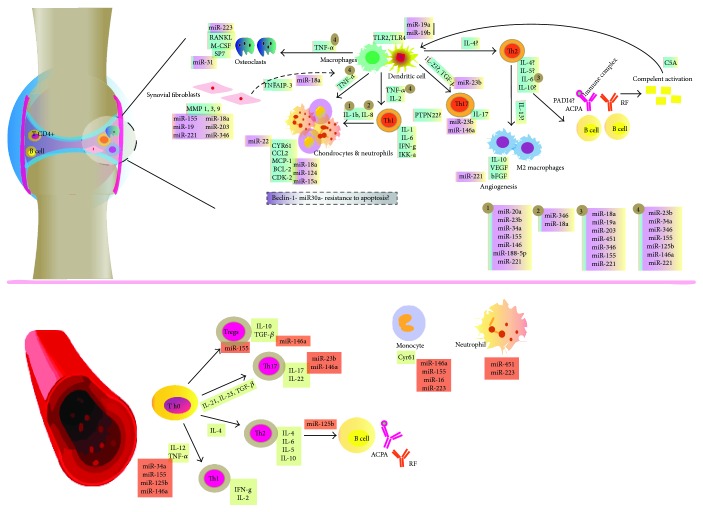
Functional role of miRNAs in rheumatoid arthritis.

We were left with 48 articles: 17 reviews and 31 original articles. We found that the identification of miRNAs was performed in different compartments such as synovial tissue and peripheral blood, and in some cases, they were not restricted to liquid phases, but researchers used selected cells to study miRNA expression including macrophages, dendritic cells (DCs), different subsets of lymphocytes, synovial fibroblasts, and osteoclasts. Most of the studies were conducted in RA patients and compared to patients with OA, and a few studies included healthy subjects as a control group. We analyzed all the information and distributed results from different studies per miRNA site of expression in peripheral or synovial tissues and then described their roles with respect to cells and effector molecules. We then compared results from miRNA dysregulation into mechanisms that are known to be related to the generation of RA and analyzed possible explanations for their participation; additionally, we pointed out specific areas where miRNAs might also have a pathogenic role but remain undescribed (Figure 2). Whenever available, we also described associations between miRNA dysregulation and clinical parameters related to RA or response to treatment.

### 3.1. miRNAs in the Periphery

#### 3.1.1. miRNAs in Peripheral Blood

Murata et al. used a TaqMan miRNA array to evaluate the expression of 768 miRNAs in peripheral blood from 102 RA patients and 104 healthy subjects; they found 26 miRNAs with significant differences in expression patterns. Ten miRNAs (let-7e, miR-128, miR-323-3p, miR-133b, miR-18b, miR-144, miR-451, miR-150, miR-486-3p, and miR-196b-5p) reflected a fourfold differential expression, and three miRNAs (miR-130b-5p, miR-452, and miR-579) were only identified in plasma from RA patients. The rest were selected due to variations in expression patterns, significant *P* value, and Ct mean value. In a second part of this study, these 26 miRNAs were evaluated in patients with different conditions. The combination of three specific miRNAs, miR-24, miR-26a, and miR-125a-5p, named as “estimated probability of RA by plasma miRNAs” (e-PRAM), was proposed to be used as potential biomarkers for the identification of RA patients, with a sensitivity and specificity of 78.4% and 92.3%, respectively [22]. Nevertheless, the most widely studied miRNAs in RA include both miR-155 and miR-146a, since they can be detected in whole blood, allowing their use to be more feasible [23]. Later, Duroux-Richard et al. focused on miR-125b, due to its involvement in the regulation of signaling pathways during inflammation, B cell differentiation, TNF production, and apoptosis. They found significant elevated levels of miR-125b in RA patients compared to healthy donors; this increase was, however, also found in patients with other chronic inflammatory arthritis such as OA [24], making evident the fact that while miRNAs are feasible to measure in peripheral blood, they still cannot differentiate between two entities with inflammatory affection of the joints. Additionally, there is a growing interest to know if circulating miRNAs can identify and distinguish early stages of RA, as they will be used for early diagnosis and as predictors of disease outcome. In this sense, one study reported reduced levels of miR-223 and miR-16 in the sera of patients with early rheumatoid arthritis (ERA) compared to those with established RA, speculating that in ERA these miRNAs may be taken up by cells where they can perform several functions, including inflammatory responses [25]. Nevertheless, new studies are required to confirm this hypothesis and prospective studies would be required to clarify their role in future stages of the diseases.

#### 3.1.2. miRNA Polymorphisms as Genetic Risk Factors for RA

As it is widely known, genetic liability is an important factor contributing to the development of RA, and recently SNPs (single-nucleotide polymorphisms) in miRNA genes have been identified to be involved in RA risk in the Caucasian population, with evidence supporting the participation of the rs3746444 polymorphism in miR-499 and RA [26]. miR-499 is involved in the regulation of genes that codify for IL-17RB, IL-23a, IL-2RB, IL-6, and IL-2. These proteins are related to several biological processes of inflammation, such as the TNF-*α* signaling pathway, or in the promotion and perpetuation of inflammatory responses [27]. El-Shal et al. confirmed that genotypes TC and CC and the allele C of the rs3746444 SNP in miR-499 are independent risk factors for joint erosions in RA. Furthermore, there was an association between the TC genotype and elevated levels of C-reactive protein (CRP), erythrocyte sedimentation rate (ESR), disease activity score (DAS-28), health assessment questionnaire (HAQ) scores, rheumatoid factor (RF), and antibodies against citrullinated peptide (anti-CCP) [28]. This association was later reproduced in an Iranian population of women with RA [27]. Additionally, miR-146 has been involved in the regulation of the genes that codify for interleukin-1 receptor-associated kinase 1 and 2 (IRAK1 and IRAK2); such proteins are involved with a functional role in toll-like receptor (TLR) signaling and cellular activation [4]. The genotype GG of rs2910164 in miR-146a was associated with RA only in females, suggesting that estrogens could lead to selective regulation of miR-146a expression in immune cells; however, further studies with larger samples and a higher diversity of ethnic groups are required to confirm such suggestions [29].

#### 3.1.3. miRNAs in Peripheral Blood and RA Treatment

A wide variety of new treatments based on biologic agents have emerged in the last decades to treat RA with the goal of achieving disease control and sustained remission; however, response to these agents is heterogeneous and difficult to predict. Based on the finding of several plasma miRNAs, the potential value of these molecules as biomarkers for therapeutic response has emerged. As miR-125b participates in the regulation of TNF production and B cell differentiation, Duroux-Richard et al. assessed the expression of this miRNA in plasma prior to treatment with rituximab and three months after, suggesting with their findings that RA patients with low expression of miR-125b at the time of disease flare are significantly less likely to improve clinically after three months of rituximab and that the overexpression of this miRNA could be useful as a predictive biomarker. Nevertheless, it is important to acknowledge that the overexpression of miR-125b is not restrictive of RA since it has been identified as well in OA [24]. In a second attempt to use miRNAs as biomarkers for treatment response, Castro-Villegas et al. examined serum samples from 10 RA patients prior to and six months after treatment with anti-TNF/DMRAD combination; by analyzing miRNA arrays, they could identify that 75 miRNAs increased and 9 miRNAs decreased their expression after treatment [1]. The detailed analysis of the altered miRNAs with the use of Ingenuity Pathway Analysis (IPA) demonstrated that target mRNAs were involved in immune and inflammatory responses, but even more interestingly, a cluster of these altered miRNAs after treatment belong to important regulators of chondrocyte maturation and proliferation. In total, 10 miRNAs were identified with a 2-fold change when comparing both times in the treatment line. From these, only six (miR-16-5p, miR-23-3p, miR-125b-5p, miR-126-3p, miR-146a-5p, and miR-223-3p) reached significance with high confidence levels, and all of them were elevated exclusively in responders to treatment. Moreover, miRNAs significantly upregulated after treatment paralleled a reduction in TNF-*α*, IL-6, IL-17, and RF. Additionally, after performing ROC curve analysis, two miRNAs were identified to have the highest area under the curve (miR-23 and miR-223) and furthermore, when analyzed in combination, demonstrated a higher sensitivity and specificity to identify responders to treatment, in relation to the identification made with other miRNAs alone. Furthermore, in general, patients with elevated levels of miRNAs before starting the therapy were nonresponders at the end of the study [1].

#### 3.1.4. miRNAs in Peripheral Blood Mononuclear Cells

Since 2008, increased levels of some miRNAs, miR-146a, miR-155, miR-132, and miR-16, have been reported in peripheral blood mononuclear cells (PMBCs) of patients with RA, and even some of these have been associated with disease activity, specially miR-146a and miR-16 [19, 26].

The interest for miR-146a has been growing in the last decade because of its major negative role in inflammation during the innate immune response [13]. Based on several *in vivo* and *in vitro* studies, there is evidence that miR-146a is an important player in the NF-*κ*B signaling pathway as it downregulates mRNAs of IL-1 receptor-associated kinase 1 (IRAK1) and tumor necrosis factor receptor-associated factor 6 (TRAF6), thereby interfering with intracellular signaling. The overexpression of miR-146a contributes to the presence of high levels of the inflammatory cytokine TNF-*α* in peripheral blood. Abou-Zeid et al. assessed the expression of miR-146a on PBMCs of patients with RA compared to patients with OA. They found that the median miR-146a expression, in these cells, was significantly higher when compared to expression patterns in cells from patients with OA. Furthermore, there was a correlation between miR-146a expression and disease activity in RA patients [5]. Additionally, another study that analyzed miR-146 in PBMCs from RA demonstrated that specific polymorphisms in IRAK1 could lead to increased susceptibility for RA, emphasizing again the importance of polymorphisms in the interaction between miRNAs and their targets [12, 30].

Experimental studies with the use of miR-155 *null* mice pointed out its role in lymphocyte differentiation and immune responses; therefore, studies in humans were conducted to try to establish its role in autoimmunity [31]. Reported targets for miR-155 include transcription factors PU.1 and c-Maf involved in hematopoietic development and dendritic cell maturation, the anti-inflammatory and proapoptotic molecule Fas-associated death domain protein (FADD), the I*κ*B kinase (IKK) that phosphorylates the inhibitory I*κ*B*α* protein, therefore positively regulating the nuclear factor-kappa B (NF-*κ*B), the receptor-interacting serine-threonine kinase 1 (RIPK1) important for perpetuating inflammatory responses, and the SH2 domain-containing inositol 5-phosphatase (SHIP-1) which inhibits proliferation of myeloid and lymphoid cells [32]. In RA, a study performed to investigate the miR-155 pattern of expression in PBMCs confirmed that it was significantly increased in RA when compared to healthy controls, although this significant difference was proved with the use of microarray experiments but not with the use of qRT-PCR; neither was the difference sustained when comparing the miR-155 expression in PBMCs from RA to OA [31]. It seems that miR-155 has a role related to an inflammatory milieu, since this same analysis in synovium gave an even higher increase in the expression of miR-155 in RA compared to both healthy and OA patients. Additionally, upon stimulation with TNF-*α*, there was evidence of miR-155 upregulation, which correlated with c-reactive protein (CRP) levels and supports its role in association with inflammatory responses. In another study, to further investigate the role of miR-155 in RA, its levels correlated with plasma levels of TNF-*α* and IL-1*β*, erythrocyte sedimentation rate (ESR), and disease activity score (DAS-28) [33]. To explain the mechanisms by which miR-155 is associated with inflammation, researchers found that suppressor of cytokine signaling 1 (SOCS1) is probably one of the most important targets of miR-155; by interacting with its 3′ UTR region, it decreases SOCS1 transcription and therefore its availability, which normally functions as a negative regulator of cytokine signaling and targets proteins for degradation by the proteasome. SOCS1 has a critical role in the regulation of IFN-*γ*, IL-4, IL-12, and IL-5 signaling. Additionally, SOCS1 is involved in T cell activation [33, 34].

As described before, miR-16 was reported to be overexpressed in PBMCs of RA patients with active disease. Additional data has been published regarding this matter, showing that higher levels of miR-16 in the plasma of patients with ERA are associated with lower disease activity during the first three months of therapy with disease-modifying antirheumatic drugs (DMARDs). Similar findings were made with miR-223, supporting the fact that these two miRNAs could characterize patients with good or bad response to therapy. This information has a high clinical significance, as most responders with ERA are seen in this period [25]. Several studies have demonstrated the participation of miR-21 in the development of inflammatory diseases such as RA and in the process of regulatory T cell differentiation. Dong et al. analyzed the expression of miR-21 in PBMCs and CD4^+^ T cells from RA patients and healthy controls; they found that the expression of miR-21 was significantly low in patients with RA. This miRNA is associated with an increase in the activation of STAT3 mRNA, a transcription factor that participates in the differentiation of Th17 cells, which are important contributors of inflammatory responses in RA [35].

Otherwise, the effect of ACPAs on the expression of miRNAs and its contribution to the inflammatory response in RA has not been investigated. However, Lai et al. assessed the expression of let-7a (one of the commonly expressed miRNAs in macrophages) in monocytes from ACPA-positive RA patients, finding that ACPAs could suppress let-7a expression levels in these cells and could to contribute to the pathogenesis of RA, as the decreased expression of this molecule could enhance the expression of Ras, which contributes to the destruction of the cartilage and bone in RA. They also demonstrated that the transfection of let-7a could suppress the mRNA and protein expression of IL-1*β*, an important proinflammatory cytokine in RA pathogenesis [36].

#### 3.1.5. miRNAs in Peripheral Neutrophils

Neutrophils are the most abundant leukocytes in human blood and play crucial roles in innate immune response. Their production is regulated by a negative feedback loop, based on their rate of apoptosis. The most important and studied physiological function of neutrophils is phagocytosis; however, their status in chronic inflammatory diseases is characterized by constant recruitment and tissue damage. Recent studies have positioned them at the center of inflammation, and even though there is still an important gap of knowledge regarding how miRNAs regulate neutrophil biology, their identification and characterization will open great opportunities for next-generation anti-inflammatory therapies. Currently, several methods are being attempted to control this inflammatory environment by targeting immune cell recruitment, neutrophil degranulation, and proinflammatory cytokine release; however, most of them are limited by important side effects [37]. Human neutrophils express 148 different miRNAs; one of the most important is miR-223, since results from several studies indicate that this miRNA is preferably expressed in the myeloid cell lineage and its level of expression positively correlates with the progression of granulopoiesis. Ismail et al. along with the work of other researchers found that miR-223 is a central mediator of an intercellular crosstalk between immune cells, as it packs itself with HDLs (high-density lipoproteins) and is transported into endothelial cells which do not transcribe miR-223 [38]. Another miRNA of interest expressed by neutrophils is miR-4661; its expression seems to rise during initiation of inflammatory response and quickly declines before neutrophil infiltration, suggesting its potential role in acute inflammation and as a potential signaling molecule. Furthermore, RA patients have reduced levels of miR-451, and there is evidence to support that restoration of its expression reduces the severity of the disease, making it an attractive candidate for therapeutics [37].

#### 3.1.6. miRNAs in Peripheral T Cells (CD4^+^, CD8^+^, and NKTs)

In the last few years, there was an interest in miRNA expression from naive CD4^+^ T cells, since they are known to play key roles in the pathogenesis of RA; these findings suggested that miRNAs could target genes implicated in lymphocyte responses and contribute to the onset of RA. Fulci et al. identified an overexpression of miR-223 in T cells and plasma from RA patients; however, results are debatable [39]. While some researchers found that the overexpression of miR-223 suppressed osteoclastogenesis, others have recently demonstrated that this overexpression could impair the production of IL-10 (a potent anti-inflammatory cytokine in RA synovial tissue) in activated T cells through targeting IGF-1R [40]. As in previous plasma analysis, the presence of miR-125b has been recently described in CD4^+^ T cells from RA patients; authors who made this finding speculated on the role of miR-125b in the preservation of naïve CD4^+^ T cells and the prevention of differentiation and acquisition of a memory phenotype [24]. Additionally, miR-451 was also found to be expressed by T cells from RA patients and to be associated with elevated DAS-28, serum ESR, and IL-6 [41].

DNA methylation is an additional epigenetic factor that contributes to the regulation of gene expression; an interesting study performed by Yang et al. analyzed CD4^+^ T cells from RA patients, along with the expression of 2 integral membrane proteins important for cell activation, which are CD11a and CD70; they found an inverse correlation between miR-126 expression and methylation patterns of *CD11a* (*ITGAL*) and *CD70* (*TNFSF7*) in CD4^+^ T cells; therefore, a higher expression of miR-126 was associated with an increased transcription of both genes [42].

#### 3.1.7. miRNAs in Peripheral Regulatory T Cells

Regulatory T cells maintain immune homeostasis through suppression of lymphocyte proliferation and cytokine production. Despite the quantity of regulatory T cells (Tregs) present in the synovial fluid of patients with RA, the inflammatory state of the joints persists, suggesting functional defects in these cells, which seem to fail the suppression of proinflammatory cytokines such as IL-17, IFN-g, and TNF-*α*. A wide variety of miRNAs have been shown to play an important role in the regulation of Tregs, but interestingly, the naïve and memory forms of these cells are characterized by different miRNA expressions [41]. It was demonstrated that the expression of miR-146a may be correlated with augmented activation of the signal transducer and activator of transcription 1 (STAT1), which leads to Treg suppression function [43]. Additionally, Zhou et al. reported a difference in miR-146a when comparing Tregs from RA patients based on their disease activity (DAS-28 score), pointing out that patients with low scores had significantly higher levels of miR-146a. This correlation was also established for tender joint count (TJC28 and TJC68) and swollen joint count (SJC28 and SJC66); however, the absence of correlation with parameters of systemic inflammation suggests that systemic inflammation appears to be less dependent on Treg function [29]. Furthermore, it was demonstrated that an increased expression of miR-155 in Tregs might favor their suppressor potential, therefore contributing to an anti-inflammatory response [44].

Figure 2 is a schematic representation of miRNA dysregulation within synovial and peripheral tissues.

During RA affection of articulations, macrophages accumulate in synovial lining and sublining, where they produce cytokines and chemokines that contribute to inflammation and cell recruitment as well as to bone erosion and cartilage destruction. The most prominent cytokine produced by macrophages is the tumor necrosis factor (TNF). Macrophages might be activated by cell contact or T cell-released cytokines such as interferon-g (IFN-g), interleukin-12 (IL-12), and interleukin-17 (IL-17), produced by Th1 and Th17 subsets of CD4^+^ T cells, respectively. Additional forms of macrophage activation include immune complexes that trigger Fcg receptor (FcgR) signaling and by molecular pattern recognition receptors, such as toll-like receptors (TLRs). Both TLR-2 and TLR-4 are highly expressed in RA synovial tissue; bacterial and endogenous ligands for TLR-4 and TLR-2 have been previously described, including Hsp96, fibrinogen, and more importantly citrullinated fibrinogen. Synovial fibroblasts (SF) are implicated in the inflammatory response by synthesizing cytokines, matrix-degrading enzymes, and other inflammatory mediators. Both SF and infiltrating T cells take part in differentiation and activation of osteoclasts by the RANK-RANK ligand (RANK-L) pathway and through the release of IL-6. Synovial fibroblasts from RA patients are known as RASF; they exhibit a much more aggressive phenotype with the capability to synthesize angiogenic factors and invade neighbor tissues.

### 3.2. miRNAs Localized in Synovial Tissues and Synovial Cells

#### 3.2.1. miRNAs in Synovial Tissues

The synovium is a membrane attached to skeletal tissue (bone-cartilage) that borders the joint cavities and lines up tendon sheaths and bursae. Normally, the synovial lining layer comprises an intimal lining formed by two or three layers of fibroblast-like synoviocytes (FLS) and macrophage-like synoviocytes embedded in a dense extracellular matrix and a sublining layer of loose connective tissue that contains blood vessels, lymph vessels, fibroblasts, collagen fibers, nerve fibers, and only very few leucocytes [45]. The development of RA requires an interplay of a variety of different immune and resident cells, characterized by an imbalance between pro- and anti-inflammatory cytokines, which leads to hyperplasia and chronic inflammation in the synovium. At the cellular level, there is activation of macrophage-like synoviocytes, proliferation of FLS, infiltration and retention of lymphocytes along with other inflammatory cells, and angiogenesis; these changes are related with later events such as the formation of an invasive pannus that destroys the articular cartilage and bone. The infiltration of lymphocytes often lacks organization; they rather express a diffuse location with T, B, and plasma cells interspersed among FLS and macrophage-like synoviocytes, which suggest their interplay. Nevertheless, in some cases (20%) lymphocytes that infiltrated the synovia are organized into large follicle germinal centers [46]; the difference in patterns might reflect the degree or time of local tissue inflammation. Furthermore, local production of autoantibodies has been reported, with human glycoprotein 39 as one identified candidate target, which was shown to be presented by the RA-associated HLA-DR4 molecule in the inflamed rheumatoid joint [47]. Additionally, several citrullinated proteins including fibrinogen can be found in inflamed joints with RA and other pathologies [48]; nevertheless, the identification of intracellular citrullinated proteins is restrictive of RA [49]. Unfortunately, the analysis of immune complexes containing citrullinated intracellular proteins and their interaction with local tissue macrophages is still missing. After analyzing gene expression profiles by means of microarray analysis in RA synovial tissue biopsies, the identification of three phenotypes was described: (1) increased expression of adaptive immune response genes (*MMP1*, *MMP3*, STAT-encoding and STAT-induced genes, and genes related to antigen presentation), (2) increased expression of genes related to extracellular matrix remodeling, and (3) a phenotype that was identical to osteoarthritis (OA) [50]. These different patterns of expression were neither associated nor correlated with clinically relevant outcomes; therefore, we still are not able to comprehend the kinetics and exact timing of immune cells and inflammatory molecule production with respect to joint affection during the disease course. In the immune and inflammatory responses, miRNAs regulate leukocyte activation and cytokine production inside joints and additionally play an important role in cartilage protection by regulating catabolic activity, proliferation, and resistance to apoptosis of synoviocytes [51]. Furthermore, the most important characteristic of miRNAs inside joints is their capability to discriminate between RA and OA, which has not been achievable in peripheral blood [52].

In the synovium, miR-124 was associated with the expression of cyclin-dependent kinase 2 (CDK2), which facilitates the secretion of MCP-1 that favors proliferation of synoviocytes [14]. Later, other studies analyzed the methylation status of miR-124a *loci* in synovial tissue from RA and OA patients and demonstrated that the gene promoter was hypermethylated only in RA, which might be associated with its downregulation [53], which was associated with excessive synoviocyte proliferation [2]. This epigenetic regulation of miR-124a in RA synovial tissue might be considered a target for DNA demethylation agents. Finally, miR-30a was found to be downregulated in synovia from RA patients; it was associated with autophagy and resistance to apoptosis, due to its interaction with the autophagy marker Beclin-1 [14].

#### 3.2.2. miRNAs in Rheumatoid Arthritis Synovial Fibroblast

Cellular lineages involved in RA include hematopoietic progenitors and mesenchymal stromal cells (MSC). Fibroblasts have MSC origin, and they can be found in bone marrow and synovial tissue. These cells can differentiate into adipocytes, osteoblasts, and chondrocytes. Synovial fibroblasts (SF) are essential to keeping the joints in shape, providing nutrients, facilitating matrix remodeling, and contributing to tissue repair; in contrast, SF isolated from RA joints, known as rheumatoid arthritis synovial fibroblasts (RASF), present an aggressive phenotype, since they proliferate during the development of the disease and participate in the accumulation, retention, and survival of leukocytes by producing a variety of cytokines, angiogenic factors, and matrix-degrading enzymes as well as displaying an invasive behavior [31]. This aggressive phenotype, documented *in vivo* and *in vitro* during early cell passages, is underpinned by epigenetic mechanisms: DNA methylation, histone modifications, and miRNA activity [54, 55]. The expression of miR-155 was investigated in synovium. Long et al. reported a 16.27-fold overexpression of miR-155 in RASF compared to osteoarthritis synovial fibroblasts (OASF). In cultured RASF, they demonstrated that silencing the expression of miR-155 can significantly promote MMP-3 production and enhance proliferation of RASF, and when transfected with a miR-155 mimic, RASF presented less secretion of MMP-3 and a less aggressive behavior; these findings made the authors suggest a possible therapeutic role for miR-155 [31]. Nevertheless, these results seem conflicting, since miR-155 is multifunctional and is involved in several immune regulatory mechanisms including B cell development, T cell-dependent antibody responses, T cell-dependent inflammatory responses, Treg suppressive functions, and activation of tissue macrophages; therefore, it is difficult to suggest its possible therapeutic effect. Furthermore, the translation from *in vitro* results to what happens *in vivo* seems challenging, since other molecules might be involved in cellular functions such as endogenous MMP-2 or MMP-9 which can contribute to RASF survival, proliferation, migration, and invasion [56]. Later, miR-221 was described as upregulated in RASF compared to OASF [57]; functionally, it was associated with increased production of MMP-9 and vascular endothelial growth factor (VEGF), which promotes angiogenesis; Yang et al. performed transfection with a miR-221 inhibitor in cultured RASF previously challenged with LPS and documented an inhibition in the production of TNF-*α*, IL-6, and IL-1*β*, which correlated with a decreased capability of RASF to migrate and invade other tissues. These findings pose another candidate for miRNAs' therapeutic role [56]. Other miRNAs upregulated in RASF and implicated with the production of MMP-1 and IL-6 are miR-346, miR-203, miR-22, and miR-18a [14]. Transfection of the latter was shown to upregulate additionally IL-8, monocyte chemoattractant protein- (MCP-) 1, and NF-*κ*B signaling by repressing the inhibitor pathway of tumor necrosis factor alpha-induced protein 3 (TNFAIP3), contributing to joint inflammation and destruction [14]. Unfortunately, we lack information to conclude weather they are part of the inflammatory response during early stages or as consequence of an ongoing inflammatory process. Furthermore, miR-188-5p was analyzed by performance of an experiment in aggressive RASF and long-time cultivated RASF (less aggressive); they were both challenged with IL-1*β* and TNF-*α*; aggressive RASF showed a 20% decreased miR-188-5p expression, after both challenges, and was associated with diminished migration patterns [55]. With respect to RASF life span, miR-34a has been identified as an apoptosis modulator and therefore with RASF lifespan. Niederer et al. analyzed the expression of miR-34a in RASF and their effects on apoptosis; they identified the X-linked inhibitor of apoptosis protein (XIAP) as a direct target of miR-34a and reported a correlation between low levels of miR-34a and high expression of XIAP in RASF and thus decreased apoptosis [58]. These results were corroborated by Chen et al., who reported a reduced basal expression of miR-34a in RASF [46]. Additionally, in this second study the expression of miR-15a was analyzed with respect to RASF apoptosis, which was found to be downregulated but contributing to apoptosis resistance, since miR-15a is a negative regulator of the expression of B cell lymphoma 2 (Bcl-2) which normally suppresses the apoptotic processes [51].

With respect to RASF activation, they can do so after ligation of TLRs; RASF express several TLRs, such as TLR1, TLR2, TLR3, TLR4, TLR5, and TLR7, although in basal conditions they only express TLR3 and TLR4 and the mRNAs of TLR2 and TLR6 [59]. From all, TLR2 and TLR-4 have been reported in RASF and synovial macrophages and significantly induced when treated with TNF-*α* or IL-1*β* [60]. Ligands for these receptors include pathogen-associated molecules but also endogenous molecules like Hsp96, fibrinogen, and specially citrullinated fibrinogen for TLR-4 [61]. As other targets, mRNA from TLR is regulated by miRNAs, and miR-19a and miR-19b were demonstrated to upregulate TLR-2 expression in RASF; even more, downregulation of miR-19b in activated RASF was associated with an increased production in IL-6 and MMP3 release, so they seem to work as negative regulators of inflammatory responses [62].

Direct targeting of RASF is recently regarded as a method for new therapies to improve the course of disease; by targeting specific miRNAs, researchers are aiming to change the RASF phenotype into a less aggressive one. Gao et al. demonstrated that miR-126 targeting *PIK3R2* can promote growth and apoptosis of RASF by regulating the PI3k/AKT signaling pathway, which was presented as a therapeutic strategy [63]. With respect to specific functions that are attributable to RASF, we can include bone resorption, which is related to the expression of Wnt proteins in RASF; the mRNA of such proteins is a target for positive regulation by miR-323, considered to be another target to modulate cellular functions in RASF [14]. de la Rica et al. performed a study to search for DNA methylation and miRNA expression in RASF and compared the results with those obtained from OA. In this study, they identified that the dysregulation in gene expression arises mainly from miRNA gene methylation; they could show 11 downregulated miRNAs located near CpG sites that were hypermethylated in RASF, including miR-124. Additionally, only 4 upregulated miRNAs were located near a CpG site hypomethylated in RASF including miR-203. This study represents a clear vision of how two different factors have intricate connections between mechanisms related to gene regulation and therefore points towards the need of investigating the multiple layers that might be associated with gene regulation in complex diseases [64].

#### 3.2.3. miRNAs in Chondrocytes

Chondrocytes also participate in the catabolic process of joints characterized by cartilage degradation and bone erosions; several pathways have been postulated for this process including the activation of Wnt/cadherin signaling, as well as a constitutive upregulation of *β*-catenin; a common component for the regulation in both pathways is miR-323. A study showed that miR-323-3p was upregulated in RA synovium compared to control subjects and, by enhancing Wnt and cadherin signaling, produced higher levels of Wnt and Fz. The activation of Wnt/*β*-catenin signaling in chondrocytes induces cartilage matrix degradation and facilitates bone erosion that contributes to the catabolic model in the bone remodeling process [65]. Another miRNA that is involved with chondrocyte proliferation is miR-23b, which regulates the mRNA that encodes a cAMP-dependent protein kinase, essential for cell proliferation. Finally, miR-140 silences ADAMSTS5, which encodes an integrin metalloproteinase, which is associated with matrix degradation in arthritic joints; therefore, miR-140 expression might be important in reducing arthritic degradation of joints [52].

#### 3.2.4. miRNAs in Osteoblasts and Osteoclasts

During osteoblast differentiation, certain miRNAs are tightly regulated including miR-30a, miR-204, miR-211, miR-320, and miR-335. They have been implicated in downregulating the runt-related transcription factor 2 (RUNX2), which normally promotes osteoblast differentiation; therefore, overexpression of these miRNAs is associated with an inhibition of differentiation to osteoblasts. Additionally, miR-31 targets CEBPA (encoding CCAAT/enhancer-binding protein a) and SP7 (zinc finger protein) in MSCs and, when overexpressed, decreases differentiation of MSC into osteoblasts or adipocytes. In the same way, miR-138 interferes with osteoblast differentiation by repression of PTK2 (focal adhesion kinase 1) [52]. In a study performed in Japan, the expression of miR-223 in RA synovium was found to be highly expressed in RA synovium when compared to OA. Furthermore, authors confirmed, *in vitro*, the capability of miR-223 to suppress osteoclastogenesis in PBMC when it was overexpressed. This suppression might be induced via RANKL or TNF-*α*; in theory, miR-223 might exert its effect in a common molecule downstream their signaling pathways. This evidence suggests that administration of miR-223 could prevent joint destruction by inhibiting osteoclastogenesis [66]. One putative target of miR-223 is repression of NF-1a, which is responsible for osteoclast differentiation and function, by upregulating the expression of the M-CSF receptor [15]. In the same way, a second study performed *in vitro* demonstrated that miR-146a was found to suppress the generation of osteoclasts; in this study, the effect was explained by reduction in mediators for osteoclast differentiation, including c-Jun, nuclear factor of activated T cells cytoplasmic 1 (NF-ATc1), PI.1, tartrate-resistant acid phosphatase (TRAP), TRAF6, and IRAK1 [67]. Thus, it is speculated that miR-146a negatively regulates osteoclastogenesis specially in an inflammatory environment [15]; therefore, its administration could prevent synovial destruction, a phenomenon that has been already proven in experimental mouse models [13].

#### 3.2.5. miRNAs in Synovial Fluid T Cells (CD4^+^)

Molecular studies on T cell repertoire in early RA established an increase in the number of highly expanded T cell clones in synovial fluid [68]. T cells are considered to play important roles in RA development; therefore, it is imperative to consider T cell regulation via miRNAs. Important subsets of T helper cells that infiltrate the synovium at early stages are Th1 cells and, during the inflammatory response Th17, play a major role, since cytokines expressed by these cells (IL-17, GM-CSF, and IL-22) are associated with synovial inflammation, mainly through their effect on neutrophil activation and through IL-17 driving of osteoclastogenesis [69]. The overproduction of IL-17 can potently suppress miR-23b, leading to overexpression of transforming growth factor- (TGB-) *β*-activated kinase 1/MAP3KT-binding protein (TAB), I*κβ* kinase- (IKK-) *α*, and TAB2 and inflammatory cytokines. The transfection of miR-23b with a small molecule (H-89) could inhibit protein kinase A (PKA) signaling and induce the differentiation of synovial fluid-derived mesenchymal stem cells (SFMSCSs) into chondrocytes with therapeutic effects on degenerative processes in RA and OA patients [14]. The inflammatory environment within arthritic joints also induces regulatory T cell (Treg) expansion, and large numbers of proliferating Tregs can be identified in these tissues whenever there is inflammation [69]. In the inflamed synovium, TNF-*α* promotes Foxp3 phosphorylation and impaired Treg function; in these conditions, Treg dysfunction correlated with T CD4^+^ cell numbers, producers of IL-17, and IFN-g. miR-21 was reported as a promoter of T helper cell (Th2) and Treg cell differentiation [35]. Furthermore, its role on sustaining Treg numbers is associated with apoptosis regulation in these cells. Recently, van der Geest et al. analyzed and compared relative levels of the expression of miR-21 and Bcl-2 transcription in Tregs from RA patients; they could demonstrate that Tregs with a memory phenotype accumulate in rheumatoid synovium with an increased expression of both Bcl-2 and miR-21 [70].

#### 3.2.6. miRNAs in Synovial Fluid CD14^+^ Cells (DCs, Macrophages, and Granulocytes)

In situ hybridization studies of RA synovial tissue revealed that miR-146a is mainly expressed by CD68^+^ macrophages and to lesser extent by IL-17-expressing T cells and CD79a B cells. Moreover, cultured RASF constitutively express high levels of miR-146a compared with OA fibroblast-like synoviocytes (OAFS) and markedly upregulate miR-146a expression upon stimulation with LPS and IL-1*β*. There is indeed a correlation between miR-146a levels and the activation of the NF-*κ*B pathway, through interaction with both IRAK (IL-associated kinase 1) and TRAFG (TNFR-associated factor 6) that modulate the IL-1-induced gene and MMP-13, which are involved in the degradation of cartilage in arthritis [15]. It would be interesting to investigate if a defective negative regulation of TRAF6 and IRAK1 in RA patients is the cause of high and prolonged TNF-*α* production. As we mentioned before, miR-155 has a proinflammatory role, since it promotes monocyte differentiation into macrophages and dendritic cells which are associated with increased TNF production after their activation. In accordance with this, miR-155 expression is higher in CD14^+^ cells isolated from synovial fluid compared to peripheral blood in RA patients [15]. Furthermore, exposure of CD14^+^ cells from healthy donors to synovial fluid from RA patients triggers an upregulation in miR-155 and induces the production of TNF-*α*, IL-6, IL-1*β*, and IL8, whereas inhibition of miR-155 suppresses TNF-*α* production [15, 32]. One of the validated targets previously described for miR-155 is SHIP-1, which inhibits proliferation of myeloid and lymphoid cells; therefore, its dysregulation probably contributes to proliferation of fibroblasts as well as infiltration of resident myeloid cells [51]. Neutrophils are also important players in RA; they get recruited after the expression of adhesion molecules and chemoattractants. Leukocyte rolling is mediated by an interaction between selectins (L-selectins), inflamed endothelial cells (E and P selectins), and P-selectin glycoprotein ligand-1 (PSLG1). Integrins (ICAM1 or VCAM1) also participate in rolling and are responsible for firm leukocyte adhesion and arrest. Chemokines participate as chemoattractants for neutrophils specially CXCL1, CXCL5, and CXCL8 [69]. The expression of miR-451 negatively regulates neutrophil migration. A high expression of miR-451 in inflamed joints correlated with elevated DAS-28 (activity score), ESR, and serum IL-6 levels [52]. These findings suggest an important role of miR-451 in modulating neutrophil recruitment and the perpetuation of an ongoing inflammatory response, therefore providing an important biomarker for both diagnosis and treatment in RA.

Table 1 summarizes major miRNAs mentioned, their genome context, and their variable changes in expression (increased or decreased) in different anatomical locations of patients with RA.

## 4. Conclusion

To unravel the causation of autoimmunity remains a big challenge for immunology, rheumatology, and genetics; important discoveries were made based on the identification of citrullinated antigens responsible for the generation of autoantibodies in rheumatoid arthritis; additionally, several biologic drugs that target inflammatory cytokines or B cell surface proteins have contributed to significant improvements in a proportion of patients with autoimmune conditions; nevertheless, up to 40% of patients do not respond favorably, therefore studies that aim in the clarification of the mechanisms that participate in the generation of an “autoimmune state” are an intensive and important field of research.

During the last two decades, miRNAs have been the object of research in several laboratories worldwide. Manifold studies have been published with respect to their role as regulators in gene expression; nevertheless, important issues remain unresolved, such as their own patterns of expression in healthy individuals and in autoimmune diseases such as RA. It is possible that changes in miRNA expression (up or down) are dependent of the immunological milieu rather than RA per se, which was evident after reviewing the lack of replication with respect to differential miRNA expression in studies from different populations. These observations confirm the pleiotropic effect that some miRNAs have depending on the cells or tissues where they are present.

Furthermore, we still do not entirely understand the biological meaning of polymorphisms or mutations in genes encoding miRNAs, how the products of miRNA gene variants interact with their mRNA targets, if this interaction is conserved if the target mRNA has polymorphisms as well, and if the presence of polymorphisms affects miRNA mechanisms of inhibition. Questions like these certainly make research around miRNAs acquire a degree of complexity that must be fully recognized.

Biomarkers are biologic parameters, objectively measured or evaluated, that provide valuable information about a physiologic or pathologic state of an individual or a population. Biomarkers could be used for screening, for risk assessment, and for evaluating responses to treatment. Ideally, biomarkers should be safe, consistent across genders or ethnics, specific, and easily determined. The identification and measurement of miRNAs are relatively easy to perform, it can be done in virtually any biological tissue or fluid, and it is a safe procedure for patients. Unresolved issues about their potential use as biomarkers include whether they are specific and consistent when analyzed under physiological scenarios but more importantly under pathological scenarios; future studies will corroborate if miRNAs are capable of fulfilling their roles as biomarkers with either predictive or diagnostic evaluation of treatment potential and therefore provide true clinical utility.

Finally, the identification of miRNAs as therapeutic agents involves other issues that should be appropriately addressed, such as multiple regulators for a single mRNA, the possibility for synergistic or antagonistic actions, and the possible participation of other regulators of gene expression including both genetic and epigenetic regulators. The analysis and thoughtfulness of these and other questions that arise from the knowledge exponentially generated in this field of biomedical research will be required, as intended in this review, to envisage the possible direction of future research that aims to describe concisely miRNAs' role in the generation of autoimmune conditions and their possibilities in becoming biomarkers or targets for treatment in RA.

## Figures and Tables

**Table 1:** Major miRNAs implicated in RA.

miRNA precursor or mature	Symbol HGNC	Genome context^†^	Gene family	miRNA site of expression (up or down) in RA		References
				Peripheral blood	Synovial tissue	
let-7e	MIRLET7E	Chr 19 [+]	let-7	P, PBMCs		[22, 36]
hsa-miR-124	MIR124-1	Chr 8 [−]	miR-124		RASF	[14, 28]
hsa-miR-124a	hsa-miR-124-3p	Chr 8 [−]	miR-124		ST	[4, 28, 53, 64]
hsa-miR-125a-5p	MIR125A	Chr 19 [+]	miR-10	P, PB		[14, 22]
hsa-miR-125b	MIR125B1	Chr 11 [−]	miR-10	P, PTc		[24]
hsa-miR-125b-5p	hsa-miR-125b-5p	Chr 11 [−]	miR-10	S		[1]
hsa-miR-126	MIR126	Chr 9 [+]	miR-126	PTc	RASF	[42, 63]
hsa-miR-126-3p	hsa-miR-126-3p	Chr 9 [+]	miR-126	P, PB, S		[1, 22]
hsa-miR-128	MIR128-1	Chr 2 [+]	miR-128	PB		[22]
hsa-miR-130b-5p	MIR130	Chr 22 [+]	miR-130	P, PB		
hsa-miR-132	MIR132	Chr 17 [−]	miR-132	P, PB, PBMCs		
hsa-miR-133b	MIR133B	Chr 6 [+]	miR-133	P, PB		
hsa-miR-138	hsa-miR-138-5p	Chr 3 [+]	miR-138		O/O	[52]
hsa-miR-140	MIR140	Chr 16 [+]	miR-140		C	[52]
hsa-miR-144	MIR144	Chr 17 [−]	miR-144	PB		[22]
hsa-miR-146a	Has-miR-146a-3p	Chr 5 [+]	miR-146	S, PBMCs, PRTc (SNP)	SFTc, SFCD14^∗^c, O/O	[2, 4, 5, 14, 15, 19, 26, 29, 30, 43, 67]
hsa-miR-146a-5p	hsa-miR-146a-5p	Chr 5 [+]	miR-146	S		[1]
hsa-miR-150	MIR150	Chr 19 [−]	miR-150	PB		[22]
hsa-miR-155	MIR155	Chr 21 [+]	miR-155	P, PBMCs, PRTc	SFCD14^∗^c, RASF	[2, 4, 14, 15, 23, 31–33, 43, 44]
hsa-miR-15a	MIR15A	Chr 13 [−]	miR-15		RASF	[51]
hsa-miR-16	MIR16-1	Chr 13 [−]	miR-15	PB, PBMCs, S		[8, 14, 25, 54]
hsa-miR-16-5p	hsa-miR-16-5p	Chr 13 [−]	miR-15	S		[1]
hsa-miR-18a	MIR18A	Chr 13 [+]	miR-17		RASF	[14]
hsa-miR-18b	MIR18B	Chr X [−]	miR-17	P, PB		[22]
hsa-miR-188-5p	hsa-miR-188-5p	Chr X [+]	miR-188		RASF	[55]
hsa-miR-193b	MIR193B	Chr 16 [+]	miR-193	PB		[22]
hsa-miR-196b-5p	hsa-miR-196b-5p	Chr 7 [−]	miR-196	PB		[22]
hsa-miR-19a	MIR19A	Chr 13 [+]	miR-19		RASF	[62]
hsa-miR-19b	MIR19B1	Chr 13 [+]	miR-19		RASF	[62]
hsa-miR-202	MIR202	Chr 10 [−]	miR-202	P, PB		[22]
hsa-miR-203	MIR203	Chr 14 [+]	miR-203		RASF	[4, 14, 64]
hsa-miR-204	MIR204	Chr 9 [−]	miR-204		O/O	[52]
hsa-miR-21	MIR21	Chr 17 [+]	miR-21	PBMCs, PRTc	SFTc	[8, 35, 70]
hsa-miR-211	MIR211	Chr 15 [−]	miR-204		O/O	[52]
hsa-miR-22	MIR22	Chr 17 [−]	miR-22		RASF	[8]
hsa-miR-221	MIR221	Chr X [−]	miR-221		RASF	[51, 52, 56]
hsa-miR-223	MIR223	Chr X [+]	miR-223	PB, PBMCS, PN, PTc, S	O/O	[1, 15, 25, 40, 54, 66]
hsa-miR-223-3p	hsa-miR-223-3p	Chr X [+]	miR-223	S		[1, 65]
hsa-miR-23	MIR23A	Chr 19 [−]	miR-23	S		[1]
hsa-miR-23-3p	hsa-miR-23a-3p	Chr 19 [−]	miR-23	S		
hsa-miR-23b	MIR23B	Chr 9 [+]	miR-23		SFTc, C	[8, 14]
hsa-miR-24	MIR24-1	Chr 9 [+]	miR-24	PB		[8, 14]
hsa-miR-26a	MIR26A1	Chr 3 [+]	miR-26	PB		[8]
hsa-miR-28-3p	hsa-miR-28-3p	Chr 3 [+]	miR-28	PB		[22]
hsa-miR-28-5p	hsa-miR-28-5p	Chr 3 [+]	miR-28	PB		
hsa-miR-30a	MIR30A	Chr 6 [−]	miR-30		ST, O/O	[8, 14]
hsa-miR-30c	MIR30C1	Chr 1 [+]	miR-30	PB		[22]
hsa-miR-30c-3p	hsa-miR-30c-1-3p	Chr 1 [+]	miR-30	PB		
hsa-miR-31	MIR31	Chr 9 [−]	miR-31		O/O	[52]
hsa-miR-320	MIR320A	Chr 8 [−]	miR-320		O/O	
hsa-miR-323	MIR323	Chr 14 [+]	miR-154		RASF, C	[22]
hsa-miR-323-3p	hsa-miR-323a-3p	Chr 14 [+]	miR-154	PB	C	[14, 52, 57]
hsa-miR-335	MIR335	Chr 7 [+]	miR-335		O/O	[52]
hsa-miR-346	MIR346	Chr 10 [−]	miR-34		RASF	[4, 14]
hsa-miR-34a	MIR34A	Chr 1 [−]	miR-34		RASF	[14]
hsa-miR-374b	MIR374B	Chr X [−]	miR-374	PB		[22]
hsa-miR-451	MIR451	Chr 17 [−]	miR-451	PN, PTc	SFCD14^∗^c	[8, 14]
hsa-miR-452	MIR452	Chr X [−]	miR-452	PB		[22]
hsa-miR-486-3p	hsa-miR-486-3p	Chr 8 [−]	miR-486	PB		[22]
hsa-miR-499	MIR499	Chr 20 [+]	miR-499	PB (SNP)		[26, 27, 30]
hsa-miR-518d-5p	hsa-miR-518d-5p	Chr 19 [+]	miR-515	PB		[22]
hsa-miR-579	MIR579	Chr 5 [−]	miR-548	PB		[22]
hsa-miR-885-5p	hsa-miR-885-5p	Chr 3 [−]	miR-885	PB		[22]

^†^[+] or [−] refers to sense or antisense DNA chain, respectively. HGNC: HUGO Gene Nomenclature Committee; PB: peripheral blood; P: plasma; S: serum; PBMCs: peripheral blood mononuclear cells; PTc (CD4^+^, CD8^+^, and NKTs): peripheral T cells; PRTc (Tregs): peripheral regulatory T cells; ST: synovial tissue; SFTc: synovial fluid T cells; SFCD14^∗^c: synovial fluid CD14^+^ T cells; DCs: dendritic cells; RASF: rheumatoid arthritis synovial fibroblast; C: chondrocytes; O/O: osteoblasts and osteoclasts; SNP: single-nucleotide polymorphism; NA: not available.

## Abbreviations

miRNA: micro-RNA
RISC: RNA-induced silencing complex
Ago2: Argonaut 2
TRBP: Human immunodeficiency virus transactivating response RNA-binding protein
IL: Interleukins
RANKL: Receptor activator of nuclear factor kappa-B ligand
TLR: Toll-like receptors
TNF-*α*: Tumor necrosis factor alpha
MMP: Matrix metalloproteinases
CCL2: Chemokine ligand 2
CDK2: Cyclin-dependent kinase 2
Cyr61: Cysteine-rich angiogenic inducer 61
TNFAIP3: Tumor necrosis factor alpha-induced protein 3
BCL2: B cell lymphoma 2
M-CSF: Macrophage colony-stimulating factor
IKK-*α*: Inhibitor of nuclear factor kappa-B kinase subunit alpha
*PADI4*: Peptidylarginine deiminase 4 gene
Tregs: Regulatory T cells
*PTPN22*: Protein tyrosine phosphatase, nonreceptor type 22 gene.
